# Assessing the precision of morphogen gradients in neural tube development

**DOI:** 10.1038/s41467-024-45148-8

**Published:** 2024-02-01

**Authors:** Marcin Zagorski, Nathalie Brandenberg, Matthias Lutolf, Gasper Tkacik, Tobias Bollenbach, James Briscoe, Anna Kicheva

**Affiliations:** 1 Institute of Theoretical Physics and Mark Kac Center for Complex Systems Research, Jagiellonian University, Lojasiewicza 11, 30-348 Krakow, Poland; 2 Institute of Bioengineering, School of Life Sciences, and School of Engineering, Ecole Polytechnique Fédérale de Lausanne, Lausanne, Switzerland; 3 Institute of Science and Technology Austria, Am Campus 1, 3400 Klosterneuburg, Austria; 4 Institute for Biological Physics, University of Cologne, Cologne, Germany; 5 Center for Data and Simulation Science, University of Cologne, Cologne, Germany; 6 The Francis Crick Institute, London, NW1 1AT UK

**Keywords:** Developmental biology, Biophysics

**arising from** R. Vetter & D. Iber *Nature Communications* 10.1038/s41467-022-28834-3 (2022)

In the developing neural tube, pattern forms in response to opposing BMP and Shh signaling gradients^1^. In a recent publication, Vetter and Iber present theoretical analysis based on which they conclude that a single morphogen gradient in the neural tube is sufficient to precisely position gene expression boundaries^2^. Here we discuss assumptions made by Vetter and Iber that limit the conclusions they reach, and address inaccuracies in their analysis. Given these limitations and existing evidence, it seems likely that both signaling gradients contribute to the precision of pattern formation in the neural tube.

In multiple systems, morphogen gradients have been studied by measuring fluorescent reporters of signaling activity^3^. A common practice is to estimate the gradient imprecision by assessing the variation in fluorescent intensity (FI) between individual embryos at every position in the tissue^4^. The positional error *σ*_*x*_ of the gradient is approximated by multiplying the variation of morphogen levels *σ*_*C*_ by the local gradient steepness ${\left|\frac{\partial C}{\partial x}\right|}^{-1}$ at that position: ${\sigma }_{x}\approx {\left|\frac{\partial C}{\partial x}\right|}^{-1}{\sigma }_{C}$. Vetter and Iber point out that different methods for estimating the local gradient steepness can produce different results. One method, numEPM, uses the spatial derivative of mean intensity at the position of interest. Another method, fitEPM, assumes that the mean gradient is exponential. In this case, the local steepness of the gradient is given by the fitted mean intensity at a position divided by the fitted exponential decay length. A third method, DEEM, estimates the positional error as the standard deviation of positions ${x}_{\theta,i}$ that correspond to a defined concentration threshold: ${\sigma }_{x}={{{{{\rm{std}}}}}}\{{x}_{\theta,i}\}$. The DEEM method is derived from the mathematical definition of positional error and hence considered to represent the most direct measure of positional error from an ensemble of gradients.

For low FI values, numEPM and fitEPM methods are influenced by how background FI is estimated and subtracted and by how data is binned and smoothed along the axis. Thus, in the tail of a gradient, the positional error estimates generated by the two methods are inexact and may differ. Vetter and Iber claim they can determine which of the two methods is correct by testing which method gives the result closest to estimating the precision of an artificial dataset consisting of an ensemble of exponential gradients using the DEEM method. This leads them to conclude that NumEPM is correct while FitEPM overestimates the positional error. However, this conclusion depends on the assumption that gradients are perfectly exponential. The cellular response to the signal and tissue heterogeneities generate gradient shapes that deviate from an exponential curve^5,6^. The poor signal-to-noise ratio in the gradient tail means that the real shape of gradients in this region cannot be reliably measured. Thus, judging the two methods by comparison to an artificial idealized dataset, which may not represent the true shape of gradients, is misleading. In other words, the performance of a method on an idealized dataset does not determine whether this method will work well on real data which may differ from the idealized dataset.

More importantly, Vetter and Iber’s analysis indicates that there is in fact very good agreement between the precision estimated by the different methods during the relevant stages of neural tube development (0–15ss, corresponding to 0–30 h). An examination of their Fig. 1E shows that the two methods produce identical precision estimates for time points 0–5ss. For 10–15ss, the estimates are also very similar and diverge only in the gradient tail: DEEM and numEPM estimate 5–6 cell diameters, fitEPM 6–8 cell diameters. These positional errors occur at distances >60% tissue length from the morphogen source for GBS-GFP and >45% for pSmad1/5.

**Fig. 1:**
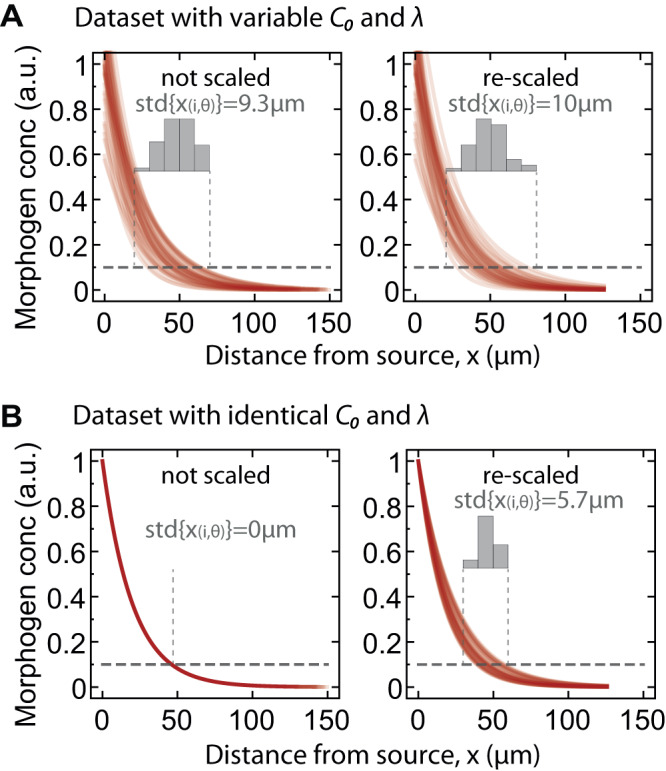
A numerical example of the rescaling error for exponential gradients with variable *C*_0_ and *λ*. **A** Left: Set of 50 randomly generated exponential morphogen profiles $C\left(x\right)={C}_{0}{e}^{-x/\lambda }$. Mean *λ* = 20 µm and *C*_0_ = 1. *λ* and *C*_0_ were varied by adding Gaussian noise with $C{V}_{\lambda }$ = 0.2 and $C{V}_{{C}_{0}}$ = 0.2. The domain length was randomly selected from uniform distribution between min *L* = 100 µm and max *L* = 150 µm. 1 cell diameter (cd) = 4.9 µm (as in^1^). Dashed horizontal line indicates a concentration threshold ${C}_{\theta }$ = 0.1. At this threshold, the histogram of positions is shown, the mean position ${{{{{\rm{mean}}}}}}\{{x}_{i,\theta }\}$ is 48.4 µm from the source, and the positional error is ${{{{{\rm{std}}}}}}\{{x}_{i,\theta }\}$ = 9.3 µm = 1.90 cd. Right: The profiles are rescaled to the average length, ${{{{{\rm{mean}}}}}}\{{L}_{i}\}$ = 125.7 µm, by rescaling each ${\lambda }_{i}$ by a factor ${{{{{\rm{mean}}}}}}\left\{{L}_{i}\right\}/{L}_{i}$. In this set, ${{{{{\rm{mean}}}}}}\{{x}_{i,\theta }\}$ = 48.8 µm, and ${{{{{\rm{std}}}}}}\{{x}_{i,\theta }\}$ = 10.0 µm = 2.05 cd. This indicates that rescaling changed the positional error estimate ${{{{{\rm{std}}}}}}\{{x}_{i,\theta }\}$ by 0.15 cd. **B** In Vetter and Iber, a scaling correction is estimated for exponential profiles without variability. A set of 50 such profiles is shown without and with rescaling (left and right, respectively). Assuming a uniform distribution of values at any given concentration threshold, the scaling error increases with distance to the source and reaches a maximum of 3 cd. Thus, the scaling error corresponds to $3\xi$, where $\xi$ denotes the relative position of the bin from the source. For the mean position at ${C}_{\theta }$ considered here (${{{{{\rm{mean}}}}}}\left\{{x}_{i,\theta }\right\}$ = 46.7 µm), $\xi$ = 46.7/125.7 = 0.37, hence the implied scaling correction is 1.11 cd ( = 5.4 μm). This is much higher than the rescaling error of 0.15 cd that we obtained for the dataset in A which incorporates realistic variability in *C*_0_ and *λ*. Note that for an opposing gradient using the same coordinate system, the scaling correction should be 3(1-*ξ*). Yet, in Fig. 2C, E of Vetter and Iber^2^, the same correction of 3(1-*ξ*) is incorrectly applied to both the GBS-GFP and pSmad gradients. Had we used 3(1-*ξ*) as in^2^, the implied correction would be 1.8 cd and be even more overestimated compared to the actual rescaling error.

The similarity in estimates at early stages are relevant, because, as we show^1^, early (before 15ss) but not late stage gradients are used to establish pattern. In^1^, we derive a decoding map of Shh and BMP signaling using the profiles measured at 5ss (Fig. 2A and S3A therein). We validate this map with experiments that are independent of how the morphogen signaling gradients were imaged. We demonstrate that the downstream transcriptional network requires morphogen input for <30 h to generate the pattern. This reinforces previous experimental evidence, based on growth rate measurements, lineage tracing and perturbation experiments, that indicates that the temporal window for morphogen-dependent cell fate specification is during the first 30 h of mouse neural tube development^7^. Thus, for the time interval relevant for pattern formation, fitEPM, NumEPM and DEEM methods produce similar estimates of positional error.

**Fig. 2:**
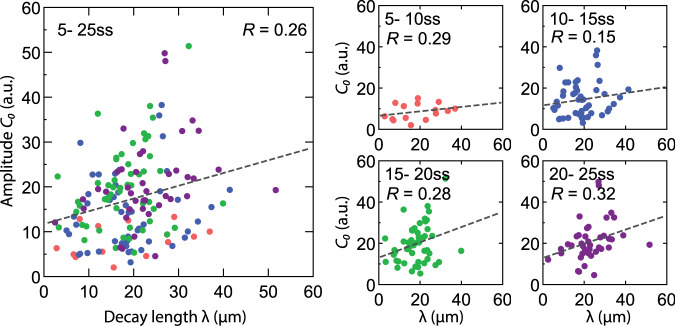
Correlation between the amplitude *C*_0_ and decay length *λ* of measured Shh gradients. *C*_0_ and *λ* are obtained from exponential fits to the measured Shh ligand gradients from Cohen et al.^11^. Here, Shh profiles were assigned to developmental stages (designated ss for somite stage) based on their DV length as described in Zagorski et al. Dashed lines are linear fits to the data. For each stage, the Pearson correlation coefficient R is shown in the plot. For the pooled set of profiles between ss5 and ss25, the correlation coefficient is 0.26. Only stages up to 25ss are shown.

Vetter and Iber also argue that imprecision of the signaling gradients is overestimated by grouping signaling profiles into bins that correspond to 10 h of developmental time. For a given bin, all signaling profiles are assumed to have the same DV length. Vetter and Iber suggest that this introduces a “scaling error”. To define it, they assume that profiles in each bin have equal amplitudes and decay lengths, but different absolute lengths. They reason that any resulting positional error is therefore the product of the differing lengths, rather than actual variability in the amplitude and decay length.

This reasoning is problematic. First, if the signaling gradient profiles are corrected in this way, so should the gene expression boundaries of Pax3 and Nkx6.1^1^. Vetter and Iber did not do this. Instead they compare the corrected signaling gradients to the imprecision of Pax3 and Nkx6.1 as reported in Zagorski et al., that is without correction.

Furthermore, by subtracting the scaling error, Vetter and Iber assume it has an additive contribution to the overall profile variability. This excludes the possibility that variability in decay length and amplitude could dominate any scaling variability. In such a scenario, subtracting the scaling error would lead to unrealistic underestimation of the actual error (Fig. 1). Taken together, the proposed “scaling error” correction is applied inconsistently and might underestimate the actual variability.

Vetter and Iber suggest that gene expression boundaries in the neural tube are positioned by a single morphogen gradient, rather than the combined interpretation of both signaling pathways. Implicit in this idea is that cells somehow distinguish which of two independent gradients is the most precise and use that to determine their identity. This interpretation also misses a crucial point: there is experimental evidence that neural progenitors respond to combinations of signaling factors. Consistent with prior studies^8^, we show^1^ that neural progenitor identities depend on the levels of both BMP and Shh signaling.

Vetter and Iber further suggest that gradient variability can be accurately inferred from “summary statistics of exponential gradients”. This necessitates several assumptions. First, gradients are assumed to be exponential. However, diffusion and degradation often depend on feedback from morphogen signaling, which can lead to deviations from exponential shape^9^. Second, ligand and signaling gradients are assumed to have comparable variability and any discrepancy results from technical measurement errors. This ignores the possibility that the signal transduction mechanisms alter the noise properties of a signal^10^. Third, variables, such as *C*_0_ and *λ*, are assumed to be independent and uncorrelated. Given that both *C*_0_ and *λ* depend on the diffusion coefficient and degradation rate, this assumption can easily be violated. Indeed assessing the correlation between *C*_0_ and *λ* for measurements taken from 5–25ss embryos reveals a modest but significant correlation of R = 0.26 (Pearson correlation coefficient; *p* = 0.001) (Fig. 2). This is inconsistent with the assumption that *C*_0_ and *λ* vary independently.

In conclusion, the assumptions inherent to the work of Vetter and Iber and their decision not to take into account experimental evidence make their conclusion, that gene expression boundaries in the neural tube are accurately positioned by a single morphogen gradient, unconvincing.

## Reporting summary

Further information on research design is available in the Nature Portfolio Reporting Summary linked to this article.

### Supplementary information

Reporting Summary

### Source data

Source Data

## Data Availability

Source data for Figs. 1, 2 is provided in the Source data file. All published data is available from us to interested researchers. Source data are provided with this paper.
